# A Genetic Approach to the Association Between *PCSK9* and Sepsis

**DOI:** 10.1001/jamanetworkopen.2019.11130

**Published:** 2019-09-11

**Authors:** QiPing Feng, Wei-Qi Wei, Sandip Chaugai, Barbara G. Carranza Leon, Vivian Kawai, Daniel A. Carranza Leon, Lan Jiang, Xue Zhong, Ge Liu, Andrea Ihegword, Christian M. Shaffer, MacRae F. Linton, Cecilia P. Chung, C. Michael Stein

**Affiliations:** 1 Division of Clinical Pharmacology, Department of Medicine, Vanderbilt University Medical Center, Nashville, Tennessee; 2 Department of Biomedical Informatics, Vanderbilt University Medical Center, Nashville, Tennessee; 3 Division of Diabetes, Endocrinology and Metabolism, Department of Medicine, Vanderbilt University Medical Center, Nashville, Tennessee; 4 Division of Genetic Medicine, Department of Medicine, Vanderbilt University Medical Center, Nashville, Tennessee; 5 Vanderbilt Genetic Institute, Vanderbilt University, Nashville, Tennessee; 6 Department of Pharmacology, Vanderbilt University, Nashville, Tennessee; 7 Division of Cardiovascular Medicine, Department of Medicine, Vanderbilt University Medical Center, Nashville, Tennessee; 8 Division of Rheumatology and Immunology, Department of Medicine, Vanderbilt University Medical Center, Nashville, Tennessee

## Abstract

**Importance:**

Whether the *PCSK9* gene is associated with the progress from infection to sepsis is unknown to date.

**Objective:**

To test the associations between *PCSK9* genetic variants, a *PCSK9* genetic risk score (GRS), or genetically estimated *PCSK9* expression levels and the risk of sepsis among patients admitted to a hospital with infection.

**Design, Setting, and Participants:**

This retrospective cohort study used deidentified electronic health records to identify patients admitted to Vanderbilt University Medical Center, Nashville, Tennessee, with infection. Patients were white adults, had a code indicating infection from the *International Classification of Diseases, Ninth Revision, Clinical Modification*, or the *International Statistical Classification of Diseases, Tenth Revision, Clinical Modification*, and received an antibiotic within 1 day of hospital admission (N = 61 502). Data were collected from January 1, 1993, through December 31, 2017, and analyzed from April 1, 2018, to March 16, 2019.

**Exposures:**

Four known *PCSK9* functional variants, a GRS for *PCSK9*, and genetically estimated *PCSK9* expression.

**Main Outcomes and Measures:**

The primary outcome was sepsis; secondary outcomes included cardiovascular failure and in-hospital death.

**Results:**

Of patients with infection, genotype information was available in 10 922 white patients for *PCSK9* functional variants (5628 men [51.5%]; mean [SD] age, 60.1 [15.7] years), including 7624 patients with *PCSK9* GRS and 6033 patients with estimated *PCSK9* expression. Of these, 3391 developed sepsis, 835 developed cardiovascular failure, and 366 died during hospitalization. None of the 4 functional *PCSK9* variants were significantly associated with sepsis, cardiovascular failure, or in-hospital death, with or without adjustment for (1) age and sex or (2) age, sex, and Charlson-Deyo comorbidities (in model adjusted for age, sex, and comorbidities, odds ratios for any loss-of function variant were 0.96 [95% CI, 0.88-1.04] for sepsis, 1.05 [95% CI, 0.90-1.22] for cardiovascular failure, and 0.89 [95% CI, 0.72-1.11] for death). Similarly, neither the *PCSK9* GRS nor genetically estimated *PCSK9* expression were significantly associated with sepsis, cardiovascular failure, or in-hospital death in any of the analysis models. For GRS, in the full model adjusted for age, sex, and comorbidities, the odds ratios were 1.01 for sepsis (95% CI, 0.96-1.06; *P* = .70), 1.03 for cardiovascular failure (95% CI, 0.95-1.12; *P* = .48), and 1.05 for in-hospital death (95% CI, 0.92-1.19; *P* = .50). For genetically estimated *PCSK9* expression, in the full model adjusted for age, sex, and comorbidities, the odds ratios were 1.01 for sepsis (95% CI, 0.95-1.06; *P* = .86), 0.96 for cardiovascular failure (95% CI, 0.88-1.05; *P* = .41), and 0.99 for in-hospital death (95% CI, 0.87-1.14; *P* = .94).

**Conclusions and Relevance:**

In this study, *PCSK9* genetic variants were not significantly associated with risk of sepsis or the outcomes of sepsis in patients hospitalized with infection.

## Key Points

**Question:**

In patients admitted to the hospital with serious infection, is there a significant association between *PCSK9* genetic variation and risk of sepsis?

**Findings:**

In this cohort study of 10 922 patients, the risk of sepsis was not significantly associated with *PCSK9* functional variants, *PCSK9* genetic risk score, or genetic estimation of *PCSK9* expression levels.

**Meaning:**

These findings suggest that *PCSK9* genetic variations are not significantly associated with the risk of sepsis in patients hospitalized with infection.

## Teaser

This cohort study tests the associations between *PCSK9* genetic variants, a *PCSK9* genetic risk score, or genetically estimated *PCSK9* expression levels and the risk of sepsis among patients admitted to a hospital with infection.

## Introduction

Sepsis is a life-threatening organ dysfunction caused by a dysregulated host response to infection^1^ and is present in 35% of deaths that occur in US hospitals.^2^ There are no specific treatments for sepsis; management consists of cardiorespiratory resuscitation and treatment of infection.^3^ Studies suggest that drugs that inhibit the *PCSK9* gene could have potential as a new treatment for sepsis.^4,5,6,7^

The *PCSK9* gene increases low-density lipoprotein cholesterol (LDL-C) concentrations by targeting LDL receptors (LDLRs) for destruction, thereby decreasing transport of LDL into the liver and thus increasing circulating LDL-C concentrations.^8,9^ Therefore, as expected, gain-of-function (GOF) and loss-of-function (LOF) variants in the *PCSK9* gene (OMIM 607786) are associated with increased and decreased LDL-C concentrations, respectively.^10^ Concordant with those observations, drugs that inhibit the *PCSK9* gene lower LDL-C concentrations markedly.^11,12,13^ However, in addition to its role in the regulation of LDL-C concentrations, *PCSK9* also appears to affect the outcomes of sepsis through LDLR-mediated effects on bacterial lipids.

Toxic lipids such as lipopolysaccharide (LPS) from gram-negative bacteria and lipoteichoic acid (LTA) from gram-positive bacteria trigger many of the manifestations of sepsis such as vasodilation, increased capillary permeability, and the production of inflammatory cytokines.^14^ Lipopolysaccharide binds to LDL-C and is taken up via LDLRs into hepatocytes, where it does not cause cell injury or cytokine production. The *PCSK9* gene plays a role in detoxifying toxic bacterial lipids such as LPS^15,16^ because it controls the number of LDLRs available.^14^ Thus, *PCSK9* could play a role in modulating responses to LPS and sepsis; several lines of evidence in animals and humans support this idea.^4,5^

In mice, deletion of *Pcsk9* attenuated cytokine and physiological responses to LPS, and treatment with a *PCSK9* inhibitor improved survival in a model of sepsis.^4^ Also, in patients with septic shock, the presence of *PCSK9* LOF variants was associated with improved survival.^4^ Consequently, drugs that inhibit *PCSK9* are under investigation as a therapy for sepsis.^5^ Moreover, because sepsis represents an extreme on a continuum of illness, the effects of *PCSK9* inhibition could be more far-reaching and may not only treat sepsis but also prevent the progression from serious infection to sepsis.

We hypothesized that *PCSK9* LOF variants protect patients with infection from developing sepsis, and, in those who did develop sepsis, that *PCSK9* LOF variants would be associated with improved in-hospital survival. We addressed this hypothesis using 3 complementary approaches: (1) *PCSK9* variants previously shown to be associated with survival in patients with sepsis^4^; (2) a *PCSK9* genetic risk score (GRS)^17^; and (3) genetically determined *PCSK9* expression levels imputed using GTEx (the Genotype-Tissue Expression project) expression quantitative traits loci data.^18,19^

## Methods

### Data Sources

Data were obtained from BioVU, a deidentified DNA biobank, and the Synthetic Derivative, a deidentified version of the electronic health record (EHR), for patients at Vanderbilt University Medical Center (VUMC). The Synthetic Derivative contains the deidentified records of approximately 2.9 million people, and their clinical information is updated every 1 to 3 months. Information available includes diagnostic and procedure codes, demographics, clinical notes, problem lists, laboratory values, and medications. BioVU is linked to the Synthetic Derivative and contains more than 285 000 DNA samples, of which approximately 87 000 have undergone genome-wide genotyping.^20,21,22^ BioVU accrues blood samples collected for clinical care that were otherwise scheduled to be discarded and applies a secure hash algorithm to deidentify the samples, which in part leads to a nonhuman subject designation with the institutional review board of VUMC. The program has received approval from the institutional review board of VUMC and was reviewed in detail by the federal Office for Human Research Protections, who agreed with the nonhuman subjects regulatory designation for the resource and subsequent research. From 2007 to 2014, BioVU operated under an opt-out accumulation model based on an opinion from the federal Office of Human Research Protection that discarded samples can be used for biomedical research without prospective consenting of each individual if the clinical data are deidentified. From 2015 to present, BioVU implemented a consented model, which adheres to the National Institutes of Health policy on genomic data sharing that was established in 2015. This study was approved by the VUMC institutional review board and followed the Strengthening the Reporting of Observational Studies in Epidemiology (STROBE) reporting guideline.

### Cohort Identification

**Figure 1.**
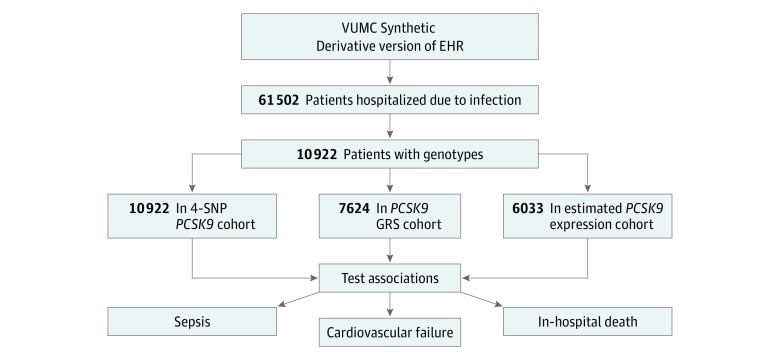
Study Design EHR indicates electronic health record; GRS, genetic risk score; SNP, single-nucleotide polymorphism; and VUMC, Vanderbilt University Medical Center.

Data were collected from January 1, 1993, through December 31, 2017, and analyzed from April 1, 2018, to March 16, 2019. Using methods previously described in detail,^23^ we identified white patients 18 years and older who had been admitted to the hospital with an infection (Figure 1) and who had genome-wide genotyping available. The day of hospital admission was designated day 0. Infection was defined as (1) having a billing code indicating infection and (2) receiving an antibiotic within 1 day of hospital admission (ie, on day −1, 0, or +1). The *International Classification of Disease, Ninth Revision, Clinical Modification* (*ICD-9-CM*) codes were used for cohort construction and covariates, and *ICD-9-CM* and the *International Statistical Classification of Diseases, Tenth Revision, Clinical Modification* (*ICD-10-CM*) codes were used for outcomes. The *ICD-9-CM* codes for infection were based on the criteria of Angus and colleagues^24^ and Donnelly and colleagues,^25^ excluding nonbacterial infections.^23^ If more than 1 qualifying episode of infection was present in a patient’s EHR, only the first was included.

### Outcomes

The primary outcome was sepsis defined using the Third International Consensus Definitions for Sepsis of concurrent infection and organ dysfunction^25^ and identified with a validated algorithm^2^ (with minor modifications^23^) that uses billing codes and clinical variables to identify sepsis in the EHR (eFigure and eMethods in the Supplement). The algorithm is highly specific (98%) and moderately sensitive (70%).^2^ Most cases of sepsis are present on admission to the hospital^2^; therefore, to minimize the confounding effects of sepsis occurring secondary to procedures or events while in the hospital, we studied sepsis occurring within 1 day of hospital admission (days −1, 0, and +1). Individuals with infection met the definition of sepsis if they had septic shock or severe sepsis or met any organ dysfunction criterion.^2,23^ Septic shock and severe sepsis were defined by *ICD-10-CM* codes that are highly specific (99%).^2^ The following criteria were used to define organ dysfunction^23^: (1) cardiovascular failure as use of the vasopressor norepinephrine bitartrate (Levophed) or the vasopressors dobutamine hydrochloride or dopamine hydrochloride and a billing code for administration of a vasopressor; (2) respiratory failure as use of codes for ventilation and admission to the intensive care unit; (3) renal failure as doubling or greater increase of baseline creatinine concentration (defined as the lowest creatinine concentration from 1 year before admission to hospital discharge); (4) hepatic failure as total bilirubin level of at least 2.0 mg/dL (to convert to micromoles per liter, multiply by 17.104) and doubled from baseline (the baseline value was defined as the lowest total bilirubin level occurring from 1 year before admission to hospital discharge); and (5) hematologic failure as platelet count of less than 100 × 10^3^/μL (to convert to ×10^9^ per liter, multiply by 1.0) and at least 50% decline from a baseline that must have been at least 100 × 10^3^/μL (the baseline value was defined as the highest platelet count occurring from 1 year before admission to hospital discharge). We also identified 2 secondary outcomes for exploratory purposes: cardiovascular failure as defined for the primary outcome (ie, requiring vasopressors) and in-hospital mortality. In-hospital death was extracted from discharge notes and death summaries. Event counts are summarized in eTable 1 and eTable 2 in the Supplement.

### Covariates

Covariates, including age and sex, were extracted from the EHR. Age was ascertained at the time of the index hospital admission. We also established the presence or absence of the comorbidities that constitute the Charlson-Deyo Comorbidity Index.^26,27,28^ We also calculated 6 principal components for ancestry using SNPRelate, version 1.16.0 (Bioconductor). For each patient, relevant diagnostic comorbidity codes occurring in the year before the index hospital admission were grouped into PheCodes^28^ using the R PheWAS package.^29^ The PheCodes were further grouped into 16 Charlson-Deyo comorbidity categories, with the categories of diabetes and diabetes with complications combined into a single diabetes variable.^23^ We previously performed a manual review of the performance of the sepsis criteria in 50 random EHRs. All patients met the inclusion and exclusion criteria, and the algorithms performed well.^23^

### Functional *PCSK9* Gene Variants and the *PCSK9* GRS

We used the following 3 approaches to examine the association between *PCSK9* gene variants and sepsis: (1) 4 *PCSK9* functional variants previously shown to affect survival in patients with sepsis^4^; (2) a *PCSK9* GRS previously shown to detect an association with risk of cardiovascular events and diabetes^30^; and (3) GTEx data to estimate genetic expression of *PCSK9*.^19^ Four *PCSK9* single-nucleotide polymorphisms (SNPs) were previously associated with sepsis outcomes^4^: rs505151 is a GOF variant, and the remainders, rs11591147, rs11583680, and rs562556, are reduced function (termed LOF) variants. Genotypes for these *PCSK9* variants were extracted for 10 922 individuals who underwent genotyping on ExomeChip or genome-wide genotype platforms with imputation through the Michigan Imputation server^31^ with minimac3, using the Haplotype Reference Consortium reference panel, version r1.1.^32,33^

We constructed the *PCSK9* GRS using the method established by Ference et al^30^ with a minor modification. Ference et al^30^ constructed a *PCSK9* GRS with 7 SNPs (rs11206510, rs2479409, rs2149041, rs2479394, rs10888897, rs7552841, and rs562556); however, rs2479409 and rs2149041 were moderately correlated (*R*^2^ > 0.3) in our cohort. Therefore, as others who have constructed *PCSK9* risk scores have also done,^34^ we removed rs2149041 and constructed a weighted 6-SNP GRS using the estimated effect size for each SNP as determined by the Global Lipids Genetics Consortium (eTable 4 in the Supplement).^35,36^

Expression of *PCSK9* in liver was imputed for individuals based on their genotypes using the expression predictors previously trained on the GTEx V6p release of RNA sequencing data (eTable 5 in the Supplement).^19^ We tested the associations between median measured LDL-C levels and (1) 4 functional *PCSK9* variants, (2) *PCSK9* GRS, and (3) estimated *PCSK9* expression (eTables 6-8 in the Supplement).

### Statistical Analysis

We used logistic regression to test the associations between *PCSK9* (4 functional variants, GRS, and genetically determined expression levels) and outcomes (sepsis, in-hospital death, and risk of cardiovascular failure). Concordant with analyses performed by others^4^ for the 4-SNP analysis, we also examined outcomes in the group of patients who carried any LOF variant. We used an additive model for single-variant association analysis and estimated odds ratios (ORs) and 95% CIs per 1-SD increase for the association between the *PCSK9* GRS or *PCSK9* expression levels and outcomes. All analyses were adjusted for (1) age and sex or (2) age, sex, and comorbidities. Models were adjusted for each comorbidity category individually because each category is likely to contribute to sepsis risk differently. To determine the association between comorbidity and outcomes, we constructed a single comorbidity score for each individual and tested the associations between this score and outcomes. Analyses were performed using PLINK, version 1.9,^37^ and R, version 3.4.4 (R Project for Statistical Computing). We conducted power calculation using principal component software (eMethods in the Supplement).^38^ The primary outcome was sepsis studied in 3 ways: (1) 4 functional *PCSK9* variants, (2) *PCSK9* GRS, and (3) genetically estimated *PCSK9* expression; thus, 2-sided *P* < .0167 was considered statistically significant. Secondary outcomes and other analyses were regarded as exploratory.

## Results

A total of 61 502 patients met the definition of infection, of whom 10 922 white adults (5294 women [48.5%] and 5628 men [51.5%]; mean [SD] age, 60.1 [15.7] years) had genotypes available for the *PCSK9* functional variants (Table 1); of these, 3391 developed sepsis, 835 developed cardiovascular failure, and 366 died during hospitalization. Mean (SD) duration of hospitalization was 7.5 (9.1) days. The comorbidity score was associated with the outcomes of (1) sepsis (OR, 1.27; 95% CI, 1.26-1.29; *P* < 2 × 10^−16^); (2) cardiovascular failure (OR, 1.29; 95% CI, 1.26-1.31; *P* < 2 × 10^−16^); and (3) in-hospital death (OR, 1.39; 95% CI, 1.37-1.42; *P* < 2 × 10^−16^) (eTable 3 in the Supplement).

**Table 1.** Demographic Characteristics of Patients With Infection and Genotyping

Characteristic	Patient Data (n = 10 922)
Sex, No. (%)	
Female	5294 (48.5)
Male	5628 (51.5)
Age, mean (SD), y	60.1 (15.7)
Comorbidities in the year preceding hospital admission, No. (%)	
Diabetes	3274 (30.0)
Any malignant neoplasm, including lymphoma and leukemia, except malignant neoplasm of skin	2831 (25.9)
Chronic pulmonary disease	2694 (24.7)
Congestive heart failure	2185 (20.0)
Cerebrovascular disease	1741 (15.9)
Metastatic solid tumor	1665 (15.2)
Myocardial infarction	1464 (13.4)
Peripheral vascular disease	952 (8.7)
Mild liver disease	867 (7.9)
Renal disease	820 (7.5)
Moderate or severe liver disease	787 (7.2)
Rheumatic disease	674 (6.2)
Peptic ulcer disease	401 (3.7)
Hemiplegia or paraplegia	301 (2.8)
Dementia	268 (2.5)
AIDS/HIV	133 (1.2)

### *PCSK9* Functional Gene Variants and Outcomes

**Figure 2.**
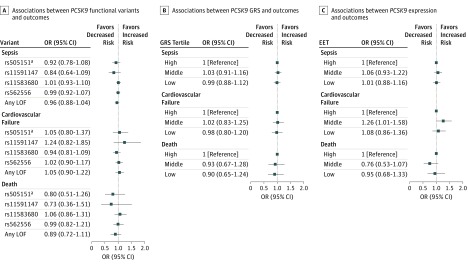
Associations Between *PCSK9* Cohorts and Sepsis Outcomes Outcomes include sepsis, cardiovascular failure, and in-hospital death. A, Associations between *PCSK9* functional gene variants and outcomes are adjusted for age, sex, and comorbidities. B and C, Associations between the *PCSK9* genetic risk score (GRS) and estimated *PCSK9* expression and outcomes are stratified by tertiles and adjusted for age, sex, and comorbidities. EET indicates estimated expression tertile; LOF loss-of-function variant; OR, odds ratio. ^a^Indicates a gain-of-function variant.

None of the 4 functional *PCSK9* gene variants were significantly associated with sepsis, cardiovascular failure, or in-hospital death (Figure 2 and eTable 9 in the Supplement) with or without adjustment for (1) age and sex or (2) age, sex, and comorbidities (in model adjusted for age, sex, and comorbidities, odds ratios for any loss-of function variant were 0.96 [95% CI, 0.88-1.04] for sepsis, 1.05 [95% CI, 0.90-1.22] for cardiovascular failure, and 0.89 [95% CI, 0.72-1.11] for death) (eTable 9 in the Supplement). The group of patients who carried any LOF variant (n = 4965) did not have outcomes that differed significantly from noncarriers (Figure 2).

### *PCSK9* GRS and Outcomes

In white patients with infection in whom genotypes were available to construct the *PCSK9* GRS (n = 7624), the GRS was not significantly associated with sepsis, in-hospital death, or cardiovascular failure in any of the models (Table 2). In the full model adjusted for age, sex, and comorbidities, the ORs were 1.01 for sepsis (95% CI, 0.96-1.06; *P* = .70), 1.03 for cardiovascular failure (95% CI, 0.95-1.12; *P* = .48), and 1.05 for in-hospital death (95% CI, 0.92-1.19; *P* = .50).

**Table 2.** Associations Between *PCSK9* Genetic Risk Score and Sepsis-Related Adverse Outcomes^a^

Phenotype	Unadjusted		Adjusted for Age and Sex		Adjusted for Age, Sex, and Comorbidities		Adjusted for Age, Sex, Comorbidities, and 6 PCs	
	OR (95% CI)	*P* Value	OR (95% CI)	*P* Value	OR (95% CI)	*P* Value	OR (95% CI)	*P* Value
Sepsis	1.01 (0.96-1.06)	.73	1.01 (0.96-1.06)	.79	1.01 (0.96-1.06)	.70	1.01 (0.96-1.06)	.70
Cardiovascular failure	1.03 (0.95-1.12)	.49	1.03 (0.95-1.11)	.55	1.03 (0.95-1.12)	.48	1.03 (0.95-1.12)	.46
In-hospital death	1.06 (0.93-1.20)	.41	1.05 (0.93-1.20)	.43	1.05 (0.92-1.19)	.50	1.05 (0.92-1.20)	.46

Abbreviations: OR, odds ratio; PCs, principal components.

^a^ Includes 7624 patients with a genetic risk score.

### Genetically Estimated *PCSK9* Expression Levels and Outcomes

In white patients with infection in whom *PCSK9* expression levels could be calculated (n = 6033), expression was not significantly associated with sepsis, in-hospital death, or cardiovascular failure in any of the models (Table 3). In the full model adjusted for age, sex, and comorbidities, the ORs were 1.01 for sepsis (95% CI, 0.95-1.06; *P* = .86), 0.96 for cardiovascular failure (95% CI, 0.88-1.05; *P* = .41), and 0.99 for in-hospital death (95% CI, 0.87-1.14; *P* = .94).

**Table 3.** Associations Between Estimated *PCSK9* Expression and Sepsis-Related Adverse Outcomes^a^

Phenotype	Unadjusted		Adjusted for Age and Sex		Adjusted for Age, Sex, and Comorbidities		Adjusted for Age, Sex, Comorbidities, and 6 PCs	
	OR (95% CI)	*P* Value	OR (95% CI)	*P* Value	OR (95% CI)	*P* Value	OR (95% CI)	*P* Value
Sepsis	1.01 (0.95-1.06)	.83	1.01 (0.96-1.06)	.76	1.01 (0.95-1.06)	.86	1.01 (0.95-1.06)	.85
Cardiovascular failure	0.97 (0.89-1.06)	.47	0.97 (0.89-1.06)	.51	0.96 (0.88-1.05)	.41	0.96 (0.88-1.05)	.41
In-hospital death	1.01 (0.88-1.15)	.94	1.00 (0.87-1.15)	.96	0.99 (0.87-1.14)	.94	1.00 (0.87-1.15)	.99

Abbreviations: OR, odds ratio; PCs, principal components.

^a^ Includes 6033 patients with estimated *PCSK9* expression.

For illustrative purposes, we show the risk of sepsis, in-hospital mortality, and risk of cardiovascular failure within different tertiles (low, middle, and high) of the *PCSK9* GRS and genetically estimated *PCSK9* expression levels. Compared with those in the highest tertile, patients in the low or middle tertiles of the *PCSK9* GRS (Figure 2 and eTable 10 in the Supplement) and of genetically estimated *PCSK9* expression levels (Figure 2 and eTable 11 in the Supplement) had a similar risk of sepsis, cardiovascular failure, and in-hospital mortality, except that the middle tertile of genetically estimated *PCSK9* expression had a higher risk of cardiovascular failure at a nominal level of significance (OR, 1.26; 95% CI, 1.01-1.58; *P* = .04).

## Discussion

The major finding of this study is that variants in the *PCSK9* gene and estimated expression of *PCSK9* were not associated with the risk of developing sepsis or the risk of poorer outcomes in patients admitted to hospital with infection. Low-density lipoprotein, through its ability to bind LPS and other toxic bacterial lipids, attenuated the severity of sepsis in animal studies.^14^ Low-density lipoprotein and its LPS cargo are internalized via LDLRs into hepatocytes, thus preventing the cellular and systemic toxic effects associated with LPS.^14^ Accordingly, because *PCSK9* increases degradation of LDLRs, it decreases LPS uptake into the hepatocytes^4,39^; thus, strategies that target the *PCSK9* gene would be expected to attenuate the toxic effects of LPS and sepsis. Indeed, 2 studies in animal models support this idea.^4,40^

Mice with a deletion of *Pcsk9* had decreased production of interleukin-6 and other inflammatory cytokines compared with control animals after they received intraperitoneal LPS; they also had attenuated physiological responses to LPS with greater preservation of mean arterial pressure and ejection fraction.^4^ In another murine model of sepsis, *Pcsk9* overexpression was associated with increased bacterial dissemination, inflammation, and histopathological damage compared with control animals, whereas mice with deletion of *Pcsk9* were protected against these effects.^40^

Pharmacological inhibition of *PCSK9* also protected against the effects of sepsis in mice; an antibody that blocked *PCSK9* blunted inflammatory cytokines and improved survival in a polymicrobial peritonitis model.^4^ These beneficial effects were absent when the gene for the LDLR was deleted, indicating that *PCSK9* inhibition altered responses to LPS through the LDLR.^4^ However, in a second study, neither *Pcsk9* deletion nor *PCSK9* antibodies protected mice against LPS-induced mortality.^41^

Less information is available to date about *PCSK9* and its association with LPS clearance and sepsis in humans. Previous observations^23,42^ found that levels of LDL-C do not appear to be directly associated with the risk of sepsis in patients with infection. In healthy participants, peak and area-under-the-curve of interleukin 6 response to an intravenous injection of LPS were attenuated in those who carried a *PCSK9* LOF allele compared with those who did not.^4^ In patients with sepsis, plasma *PCSK9* concentrations were elevated compared with those without sepsis and were associated with decreased clearance of endotoxin.^43^ Moreover, in 2 cohorts of patients with septic shock, Walley and colleagues^4,42^ reported that the groups that carried any of 3 *PCSK9* LOF variants had improved survival compared with the groups with no variants or a GOF variant. Conversely, groups with a GOF variant had worse survival than those with no variants or an LOF variant.^4^

Because sepsis is a continuum of severe illness after infection, and because studies in animals showed that *PCSK9* was associated with not only survival after sepsis but also bacterial dissemination and physiological responses to LPS and infection,^4,40^ we hypothesized that *PCSK9* decreased-function genetic variants would be associated with a lower risk of progression from infection to sepsis. We used 3 approaches. First, we studied the same 4 variants that were previously associated with altered survival in patients with septic shock.^4^ Second, we used a *PCSK9* GRS (derived from the effect of variants on LDL-C levels) that previously detected associations between *PCSK9* and risk of cardiovascular events and diabetes.^30^ Third, we estimated genetic *PCSK9* expression levels using GTEx information. The 3 approaches yielded concordant results: *PCSK9* variants were not significantly associated with risk of developing sepsis in patients with infection. Similarly, these variants were not significantly associated with the other outcomes.

Our study had several differences from that of Walley and colleagues^4^ that may account for the different conclusions. First, their patients had septic shock and were enrolled from 2 clinical trials; second, they included several ethnic groups with statistical adjustment for ancestry; and third, the outcome was 28-day mortality. The present study included not only patients with septic shock but also those with other manifestations of sepsis. Because we included all patients with sepsis at a large tertiary care hospital who had genotype information, the characteristics of these patients differ from those enrolled in clinical trials for septic shock. For example, to better establish the progressive association between infection and sepsis, we required that sepsis occur within 1 day of hospitalization for infection. Thus, patients admitted for a surgical procedure who subsequently developed sepsis in the hospital would not be included in our study. Also, we studied only white patients to minimize the effects of population stratification on any association between genotype and outcomes; however, this is unlikely to lead to a false-negative result because the sample size was large. In-hospital mortality was a secondary outcome because it is more clearly linked with the episode of sepsis, and we found no association between carriage of *PCSK9* LOF or GOF variants and this outcome. Nevertheless, we cannot exclude the possibility that beneficial effects of *PCSK9* variants on sepsis outcomes would be observed after patients are discharged from hospital.^44^

### Strengths and Limitations

The study included patients who are representative of those admitted to a tertiary care hospital with infection, and the findings are therefore broadly generalizable to that population. However, we cannot exclude a potential effect of *PCSK9* on outcomes in patients with sepsis associated with specific causes, or those with specific manifestations of sepsis. Second, information in the EHR linked to genotypes allowed us to perform the study efficiently; however, we did not perform a randomized clinical trial. One major advantage of randomization is to minimize unequal distribution of confounding variables among groups of interest. The genetic approaches used also have this advantage because constructing groups according to genotype should be minimally affected by unequal distribution of confounders. Third, we used 3 genetic approaches: the 4-SNP approach that previously found an association with sepsis outcomes,^4^ a *PCSK9* GRS previously associated with cardiovascular outcomes,^30^ and genetically estimated gene expression levels.^19^ In BioVU, rs11591147 and rs505151 and the *PCSK9* GRS were significantly associated with measured LDL-C; however, estimated *PCSK9* expression was not (eTables 6-8 in the Supplement). Protein function could be more important than variability in gene expression in regulating LDL-C levels. Furthermore, although these approaches have previously been successful in other studies, the genetic signal may have been too small to affect the outcomes, and a clinical trial of a drug that inhibits *PCSK9* in patients with sepsis could reach different conclusions. Fourth, we did not evaluate the association between statin use and risk of sepsis. The question is complex: higher LDL-C levels have been postulated to be protective in sepsis (although a previous study of LDL-C in sepsis^23^ did not support that idea), and statins, which lower LDL-C levels, have also been postulated to be beneficial in sepsis. The results have been inconclusive, and a systematic review and meta-analysis of randomized trials^45^ concluded that statin treatment was not associated with reduced mortality in sepsis. In addition, this study will require replication. The current algorithm included *International Classification of Disease* or *Current Procedural Terminology* codes, medications, laboratory test results, and knowledge of temporal associations; such information is not available in many databases. We believe that in the future, the algorithm can be modified and applied to other practice-based cohorts to test the generalizability of our observations.

## Conclusions

In this study, genetic variants of *PCSK9* were not significantly associated with the risk of sepsis or with the outcomes of sepsis in patients hospitalized with infection. We believe that future studies should modify the algorithm used and include practice-based cohorts.

## Notes

Supplement. **eMethods.** Cohort Identification and Power Calculations **eTable 1.** Event Counts in Each Analysis **eTable 2.** Event Counts by Genotypes **eTable 3.** Associations Between Comorbidity Score and Sepsis and Related Outcomes **eTable 4.** SNPs Included in *PCSK9* Genetic Risk Score **eTable 5.** SNPs Included in Estimated *PCSK9* Expression **eTable 6.** Associations Between Median Measured LDL-C Levels and 4 Functional *PCSK9* Variants **eTable 7.** Associations Between Median Measured LDL-C Levels and *PCSK9* GRS **eTable 8.** Associations Between Median Measured LDL-C Levels and *PCSK9* Expression **eTable 9.** Associations Between *PCSK9* Candidate SNPs and Sepsis and Related Adverse Outcomes **eTable 10.** Associations Between *PCSK9* GRS and Sepsis and Related Adverse Outcomes **eTable 11.** Associations Between Genetically Estimated *PCSK9* Expression Tertiles and Sepsis and Related Adverse Outcomes **eFigure.** Algorithm to Identify Sepsis Within Infection Cohort **eReferences.**
