# Supplementary material for: A Genetic Approach to the Association Between PCSK9 and Sepsis
Source: JAMA Netw Open. 2019 Sep 11;2(9):e1911130. doi: 10.1001/jamanetworkopen.2019.11130 (PMC6739725; doi:10.1001/jamanetworkopen.2019.11130)
Supplement: Supplement. — eMethods. Cohort Identification and Power Calculations eTable 1. Event Counts in Each Analysis eTable 2. Event Counts by Genotypes eTable 3. Associations Between Comorbidity Score and Sepsis and Related Outcomes eTable 4. SNPs Included in PCSK9 Genetic Risk Score eTable 5. SNPs Included in Estimated PCSK9 Expression eTable 6. Associations Between Median Measured LDL-C Levels and 4 Functional PCSK9 Variants eTable 7. Associations Between Median Measured LDL-C Levels and PCSK9 GRS eTable 8. Associations Between Median Measured LDL-C Levels and PCSK9 Expression eTable 9. Associations Between PCSK9 Candidate SNPs and Sepsis and Related Adverse Outcomes eTable 10. Associations Between PCSK9 GRS and Sepsis and Related Adverse Outcomes eTable 11. Associations Between Genetically Estimated PCSK9 Expression Tertiles and Sepsis and Related Adverse Outcomes eFigure. Algorithm to Identify Sepsis Within Infection Cohort eReferences. [file jamanetwopen-2-e1911130-s001.pdf]

Supplementary Online Content

Feng Q, Wei W-Q, Chaugai S, et al. A genetic approach to the association between *PCSK9* and sepsis. *JAMA Netw Open*. 2019;2(9):e1911130. doi:10.1001/jamanetworkopen.2019.11130

eMethods. Cohort Identification and Power Calculations

eTable 1. Event Counts in Each Analysis

eTable 2. Event Counts by Genotypes

eTable 3. Associations Between Comorbidity Score and Sepsis and Related Outcomes

eTable 4. SNPs Included in *PCSK9* Genetic Risk Score

eTable 5. SNPs Included in Estimated *PCSK9* Expression

eTable 6. Associations Between Median Measured LDL-C Levels and 4 Functional *PCSK9* Variants

eTable 7. Associations Between Median Measured LDL-C Levels and *PCSK9* GRS

eTable 8. Associations Between Median Measured LDL-C Levels and *PCSK9* Expression

eTable 9. Associations Between *PCSK9* Candidate SNPs and Sepsis and Related Adverse Outcomes

eTable 10. Associations Between *PCSK9* GRS and Sepsis and Related Adverse Outcomes

eTable 11. Associations Between Genetically Estimated *PCSK9* Expression Tertiles and Sepsis and Related Adverse Outcomes

eFigure. Algorithm to Identify Sepsis Within Infection Cohort

eReferences.

This supplementary material has been provided by the authors to give readers additional information about their work.

eMethods. Cohort Identification and Power Calculations

Cohort identification

Sepsis was defined as concurrent infection and organ dysfunction occurring within one day of hospital admission (days -1, 0, and +1) using an algorithm to detect sepsis using EHR data[1] with minor modifications. The modifications were as follows: in the definition of sepsis we included (1) septic shock defined by presence of ICD9 codes 995.92 and 785.52, or ICD10 codes R65.20 and R65.21 because these codes are very specific; [1] (2) vasopressor initiation identified by use of levophed (noprepinephrine bitartrate), or use of dobutamine or dopamine and a billing code for administration of a vasopressor (ICD9-CM procedure code 00.17 or ICD10-PCS procedure codes 3E033XZ, 3E043XZ, 3E053XZ and 3E063XZ) because dobutamine or dopamine alone had low specificity for identifying patients in whom it was used as a pressor; (3) we did not use serum lactate levels as a criterion because they were seldom available; (4) we excluded individuals who had scheduled cardiothoracic surgery because the algorithms did not reliably identify the reason for artificial ventilation or ICU admission and some of these patients had an infection and received an antibiotic. The methods used have been described in detail previously.[2]

Associations between LDL-C and (1)4 PCSK9 functional variants (2) PCSK9 GRS and (3) predicted PCSK9 expression

For LDL-C analyses (other than the gene expression analysis), we did not restrict our analysis of the relationships between the genetic instruments and LDL-C to the sepsis cohort (n=10922) because some patients may only have had LDL-C measured after hospital admission (or during

© 2019 Feng Q et al. *JAMA Network Open*

sickness) which would confound the association test. Instead, we used all available data in BioVU for individuals with LDL-C and PCSK9 genotypes. We have used all BioVU individuals who had both PCSK9 genotypes and an LDL-C measurement. We used the median LDL-C for those with multiple measurements.

The number of individuals in each analysis varies due to data availability in BioVU. Specifically, the 4 functional PCSK9 variants were extracted from genome-wide platforms and the ExomeChip (N=22,995). The 6 SNPs for PCSK9 GRS were not available on the ExomeChip and could not be imputed from it. Therefore, the association between the PCSK9 GRS and measured LDL-C was evaluated within those on genome-wide platforms (n=15,387). For the association between estimated PCSK9 expression and LDL-C, we used the sepsis cohort because gene expression had been calculated in this cohorts. Within the sepsis cohort, 3630 individuals had both predicted PCSK9 expression and measured LDL-C.

Power calculations

There was adequate power to detect small differences between the study groups; the detectable odds ratio for sepsis in the loss-of-function (LOF) carriers relative to non-carriers was 1.15. We estimated this detectable difference as a post-hoc calculation using PC software[3] and the following assumptions: (1) 4,965 patients had >= one LOF PCSK9 variant; (2) 5,162 patients did not have any functional variants; and (3) 3,391 patients developed sepsis (31%). This estimation used an uncorrected chi-squared statistic to evaluate the null hypothesis and power of 0.84. The type I error probability associated with this test of the null hypothesis was 0.05. The figure below illustrates power for a range of true ORs for current cohort.

Patients who were gain-of-function carriers were excluded from this power calculation.

© 2019 Feng Q et al. *JAMA Network Open*

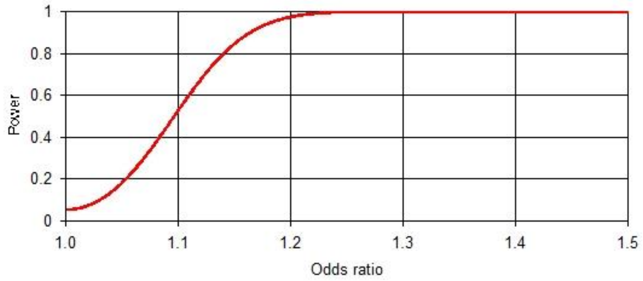

Figure. statistical power in current study

We adopted the PCSK9 GRS based on LDL-C levels from previous high impact publications.[4,5] Specifically, the four functional PCSK9 variants were used in Walley's paper in Science Translation Medicine.[4] Although there was no quantification of PCSK9's effect for removal of LPS in vivo, the same genetic instruments demonstrated the relationship between PCSK9 variants and mortality in a cohort of ~500 individuals with septic shock. Furthermore, the GRS used in our manuscript was also significantly associated with both myocardial infarction and type II diabetes mellitus. [5]

© 2019 Feng Q et al. *JAMA Network Open*

eTable 1. Event Counts in Each Analysis

Predictor	Outcomes	Gender	
		F	M
PCSK9 functional variants (4 SNPs)	sepsis	1485	1906
	cardiovascular failure	308	527
	In hospital death	148	218
PCSK9 GRS	sepsis	1015	1456
	cardiovascular failure	222	424
	In hospital death	90	149
PCSK9 expression	sepsis	826	1152
	cardiovascular failure	189	337
	In hospital death	76	131

© 2019 Feng Q et al. *JAMA Network Open*

eTable 2. Event Counts by Genotypes

		sepsis				cardiovascular failure				inhospital death			
	N of minor allele	No	Yes	No	Yes	No	Yes	No	Yes	No	Yes	No	Yes
		Counts		percent (%)		Counts		percent (%)		Counts		percent (%)	
rs11591147_T	0	7309	3299	68.9	31.1	9803	805	92.4	7.6	10250	358	96.6	3.4
	1	214	86	71.3	28.7	272	28	90.7	9.3	292	8	97.3	2.7
rs11583680_T	0	5591	2514	69.0	31.0	7471	634	92.2	7.8	7832	273	96.6	3.4
	1	1777	793	69.1	30.9	2390	180	93.0	7.0	2489	81	96.8	3.2
	2	152	73	67.6	32.4	205	20	91.1	8.9	213	12	94.7	5.3
rs562556_G	0	5168	2314	69.1	30.9	6920	562	92.5	7.5	7229	253	96.6	3.4
	1	2112	971	68.5	31.5	2835	248	92.0	8.0	2986	97	96.9	3.1
	2	247	103	70.6	29.4	325	25	92.9	7.1	334	16	95.4	4.6
rs505151_G	0	6998	3165	68.9	31.1	9390	773	92.4	7.6	9817	346	96.6	3.4
	1	512	221	69.8	30.2	671	62	91.5	8.5	713	20	97.3	2.7
	2	18	4	81.8	18.2	22	0	100.0	0.0	22	0	100.0	0.0

© 2019 Feng Q et al. *JAMA Network Open*

eTable 3. Associations Between Comorbidity Score and Sepsis and Related Outcomes

	Odds Ratio	95% confidence interval		P-value
**Sepsis**	1.27	1.26	1.29	<2e-16
**Cardiovascular Failure**	1.29	1.26	1.31	<2e-16
**Death**	1.39	1.37	1.42	<2e-16

© 2019 Feng Q et al. *JAMA Network Open*

eTable 4. SNPs Included in *PCSK9* Genetic Risk Score

SNPs	minor allele	effectsize mg/dL
rs2479394	G	1.2352
rs11206510	C	-2.6592
rs2479409	G	2.0544
rs10888897	T	-1.6224
rs7552841	T	1.1776
rs562556	G	-2.048

(Ference, B. A. *et al.* Variation in PCSK9 and HMGCR and Risk of Cardiovascular Disease and Diabetes. *N. Engl. J. Med.* **375**, 2144–2153 (2016).)

© 2019 Feng Q et al. *JAMA Network Open*

eTable 5. SNPs Included in Estimated *PCSK9* Expression

POS	ID	WEIGHT	ref_allele	eff_allele
1:54677786-54677786	rs17392549	0.04947	G	A
1:56132837-56132837	rs116532018	-0.07378	A	C
1:56073566-56073566	rs112931677	-0.02218	T	C
1:55524601-55524601	rs565436	0.069746	G	A
1:54532687-54532687	rs61777599	-0.11534	A	G
1:54637417-54637417	rs682705	-2.05E-05	G	A
1:55789748-55789748	rs71637889	0.006645	G	A
1:54654129-54654129	rs4244643	-0.05393	G	T
1:54994972-54994972	rs6675210	0.140809	T	C
1:54803850-54803850	rs66916204	-0.10365	A	C
1:54535919-54535919	rs72664136	0.155025	G	A
1:56413577-56413577	rs56375406	-0.20066	G	A
1:55522674-55522674	rs11587071	-0.01967	C	T
1:55442357-55442357	rs12062838	-0.01175	A	G
1:54632658-54632658	rs113535797	0.064701	G	A
1:55519015-55519015	rs639750	0.021681	G	T
1:54572175-54572175	rs12046178	0.01663	T	C
1:55757418-55757418	rs35818377	0.009532	A	G
1:55720674-55720674	rs12039195	-0.08535	C	T
1:55518166-55518166	rs625619	0.045018	G	A
1:54566310-54566310	rs6694397	0.017712	G	A
1:54762343-54762343	rs12118138	-0.14263	C	T
1:55647272-55647272	rs11206521	-0.09513	T	C
1:55757519-55757519	rs34262463	0.0363	T	C
1:54660252-54660252	rs148520561	0.01326	C	T
1:55785774-55785774	rs17111909	0.038863	A	G
1:55431601-55431601	rs1076528	0.02762	A	G
1:55246035-55246035	rs12144319	-0.02505	T	C
1:56170178-56170178	rs72671422	-0.14367	G	A
1:55115501-55115501	rs1655523	-0.0922	T	G
1:54557290-54557290	rs12046885	0.017157	C	T
1:55518752-55518752	rs7552841	0.194478	C	T
1:55789351-55789351	rs17111925	0.010158	G	A
1:56187341-56187341	rs2986585	0.065122	A	G
1:55366346-55366346	rs4424550	-0.026	T	C

© 2019 Feng Q et al. *JAMA Network Open*

eTable 6. Associations Between Median Measured LDL-C Levels and 4 Functional *PCSK9* Variants

CHR	SNP	BP	A1	BETA	STAT	P
1	rs11591147	55505647	T	-13.03	-1.02E+01	2.87E-24
1	rs11583680	55505668	T	-0.1629	-0.3759	0.707
1	rs562556	55524237	G	-0.6824	-1.75E+00	0.08027
1	rs505151	55529187	G	1.633	2.005	0.04499

© 2019 Feng Q et al. *JAMA Network Open*

eTable 7. Associations Between Median Measured LDL-C Levels and *PCSK9* GRS

predictor	Estimate	Std. Error	t value	Pr(>|t|)
PCSK9 GRS (n=15387)	0.7983	0.2593	3.079	0.00208

© 2019 Feng Q et al. *JAMA Network Open*

eTable 8. Associations Between Median Measured LDL-C Levels and *PCSK9* Expression

predictor	Estimate	Std. Error	t value	Pr(>|t|)
predicted PCSK9 expression (n=3630)	-0.07969	0.53808	-0.148	0.882

© 2019 Feng Q et al. *JAMA Network Open*

eTable 9. Associations Between *PCSK9* Candidate SNPs and Sepsis and Related Adverse Outcomes

CHR	SNP	Location	Minor Allele Frequency	Minor Allele	Amino acid change	Sepsis		Cardiovascular Failure		Death	
						Odds Ratio	P	Odds Ratio	P	Odds Ratio	P
**unadj.**											
1	rs11591147	55505647	0.013	T	p.Arg46Leu	0.8903 (0.6909-1.147)	0.3694	1.254 (0.844-1.862)	0.2628	0.7844 (0.3856-1.596)	0.5028
1	rs11583680	55505668	0.14	T	p.Ala53Val	1.004 (0.9249-1.091)	0.9154	0.9411 (0.8129-1.09)	0.417	1.042 (0.8457-1.285)	0.6974
1	rs562556	55524237	0.17	G	p.Ile474Val	1.004 (0.9314-1.082)	0.9156	1.039 (0.9125-1.182)	0.5657	1.021 (0.8426-1.237)	0.8321
1	rs505151	55529187	0.036	G	p.Gly670Glu	0.927 (0.7936-1.083)	0.3386	1.048 (0.8067-1.361)	0.7262	0.759 (0.4857-1.186)	0.2262
	Any LOF	-	-	-	-	0.9667 (0.8886-1.0516)	0.43	1.0489 (0.9055-1.2150)	0.524	0.8908 (0.7180-1.1041)	0.292
**adj. gender and sex**											
1	rs11591147	55505647	0.013	T	p.Arg46Leu	0.8922 (0.692-1.15)	0.379	1.261 (0.8482-1.875)	0.2516	0.7835 (0.3846-1.596)	0.5017
1	rs11583680	55505668	0.14	T	p.Ala53Val	1.006 (0.9263-1.093)	0.8837	0.9443 (0.8154-1.094)	0.4447	1.046 (0.8489-1.29)	0.6715
1	rs562556	55524237	0.17	G	p.Ile474Val	1.008 (0.9345-1.086)	0.8441	1.046 (0.9185-1.191)	0.498	1.026 (0.8468-1.244)	0.7919
1	rs505151	55529187	0.036	G	p.Gly670Glu	0.9269 (0.7933-1.083)	0.339	1.052 (0.8094-1.368)	0.7038	0.771 (0.4921-1.208)	0.2562
	Any LOF	-	-	-	-	0.9707 (0.8921 - 1.0561)	0.4894	1.0611 (0.9157 - 1.2296)	0.43	0.9077 (0.7311 - 1.1257)	0.3785
**adj. age, sex and comorbidity groups**											
1	rs11591147	55505647	0.013	T	p.Arg46Leu	0.8374 (0.6441-1.089)	0.1851	1.235 (0.8237-1.851)	0.3072	0.734 (0.3571-1.509)	0.4003
1	rs11583680	55505668	0.14	T	p.Ala53Val	1.007 (0.9252-1.097)	0.8648	0.9351 (0.8053-1.086)	0.3786	1.063 (0.8605-1.314)	0.57
1	rs562556	55524237	0.17	G	p.Ile474Val	0.9912 (0.9171-1.071)	0.8245	1.024 (0.8971-1.169)	0.7264	0.9907 (0.8147-1.205)	0.9257
1	rs505151	55529187	0.036	G	p.Gly670Glu	0.917 (0.7811-1.077)	0.2896	1.048 (0.8012-1.371)	0.7326	0.7973 (0.5061-1.256)	0.3284
	Any LOF	-	-	-	-	0.9555 (0.8759-1.0423)	0.3047	1.0461 (0.9002-1.2157)	0.5566	0.8919 (0.7158-1.1102)	0.3066
**adj. age, sex, comorbidity groups and 6PCs**											
1	rs11591147	55505647	0.013	T	p.Arg46Leu	0.8654 (0.6594-1.136)	0.2976	1.334 (0.887-2.005)	0.1664	0.817 (0.3965-1.683)	0.5836
1	rs11583680	55505668	0.14	T	p.Ala53Val	1.01 (0.9239-1.05)	0.8241	0.9356 (0.8012-1.092)	0.3997	1.063 (0.8513-1.326)	0.5914
1	rs562556	55524237	0.17	G	p.Ile474Val	0.9979 (0.9195-1.083)	0.9601	1.037 (0.9038-1.189)	0.6054	1.016 (0.8282-1.246)	0.8802
1	rs505151	55529187	0.036	G	p.Gly670Glu	0.9184 (0.7765-1.086)	0.3201	1.018 (0.7696-1.346)	0.9014	0.6528 (0.3921-1.087)	0.1011
	Any LOF	-	-	-	-	0.9642 (0.8801-1.0564)	0.4341	1.0666 (0.9127-1.2465)	0.4175	0.9128 (0.7258-1.1470)	0.4343

* rs505151 is a gain-of-function variant; other SNPs are loss-of-function variants

© 2019 Feng Q et al. *JAMA Network Open*

eTable 10. Associations Between *PCSK9* GRS and Sepsis and Related Adverse Outcomes

PCSK9 Tertiles	Sepsis		Cardiovasular Failure		Death	
	Odds Ratio	P	Odds Ratio	P	Odds Ratio	P
**unadj.**						
Low	0.9944 (0.8839-1.1188)	0.926	0.9868 (0.8089-1.2038)	0.896	0.8840 (0.6446-1.2103)	0.442
Middle	1.0424 (0.9269-1.1722)	0.489	1.0331 (0.8485-1.2581)	0.746	0.9084 (0.6638-1.2413)	0.546
High	1	-	1	-	1	-
**adj. age, sex and comorbidity groups**						
Low	0.9892 (0.8759-1.1172)	0.86165	0.9790 (0.7989-1.1997)	0.838029	0.9012 (0.6534-1.2410)	0.52436
Middle	1.0285 (0.9110-1.1611)	0.64959	1.0191 (0.8332-1.2468)	0.853655	0.9285 (0.6744-1.2766)	0.6478
High	1	-	1	-	1	-
**adj. age, sex, comorbidity groups, and 6PCs**						
Low	0.9884 (0.8750 - 1.1165)	0.85112	0.9739 (0.7944-1.1939)	0.799039	0.8879 (0.6428-1.2243)	0.46866
Middle	1.0275 (0.9100 - 1.1602)	0.66128	1.0179 (0.8320-1.2456)	0.862745	0.9202 (0.6678-1.2665)	0.60988
High	1	-	1	-	1	-

© 2019 Feng Q et al. *JAMA Network Open*

eTable 11. Associations Between Genetically Estimated *PCSK9* Expression Tertiles and Sepsis and Related Adverse Outcomes

PCSK9 Tertiles	Sepsis		Cardiovascular Failure		Death	
	Odds Ratio	P	Odds Ratio	P	Odds Ratio	P
**unadj.**						
Low	0.9977 (0.8742-1.1387)	0.973	1.0618 (0.8468-1.3318)	0.6036	0.9318 (0.6693-1.2957)	0.674
Middle	1.0674 (0.9359-1.2173)	0.331	1.2792 (1.0289-1.5927)	0.0271	0.7830 (0.5533-1.1032)	0.164
High	1	-	1	-	1	-
**adj. age, sex and comorbidity groups**						
Low	1.0078 (0.8789-1.1556)	0.911203	1.0816 (0.8583-1.3635)	0.506235	0.9492 (0.6772-1.3290)	0.76145
Middle	1.0608 (0.9259-1.2155)	0.394932	1.2637 (1.0112-1.5814)	0.040103	0.7558 (0.5305-1.0722)	0.11809
High	1	-	1	-	1	-
**adj. age, sex, comorbidity groups and 6PCs**						
Low	1.0071 (0.8782-1.1550)	0.919352	1.0793 (0.8561-1.3613)	0.518633	0.9328 (0.6643-1.3080)	0.68657
Middle	1.0621 (0.9269-1.2171)	0.38602	1.2676 (1.0140-1.5868)	0.037783	0.7496 (0.5256-1.0646)	0.10882
High	1	-	1	-	1	-

© 2019 Feng Q et al. *JAMA Network Open*

eFigure. Algorithm to Identify Sepsis Within Infection Cohort

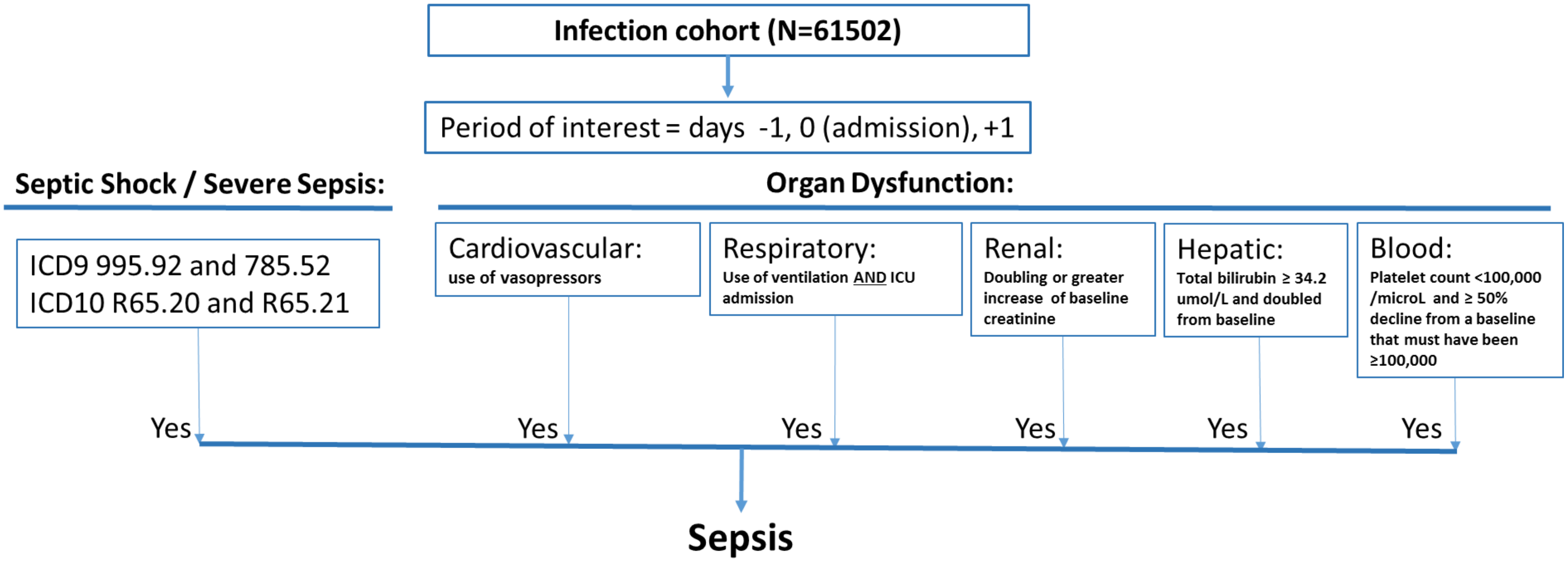

© 2019 Feng Q et al. *JAMA Network Open*

eReferences.

1. Rhee C, Dantes R, Epstein L, et al. Incidence and Trends of Sepsis in US Hospitals Using Clinical vs Claims Data, 2009-2014. *JAMA*. 2017;318(13):1241-1249. doi:10.1001/jama.2017.13836

2. Feng Q, Wei W-Q, Chaugai S, et al. Association Between Low-Density Lipoprotein Cholesterol Levels and Risk for Sepsis Among Patients Admitted to the Hospital With Infection. *JAMA Netw Open*. 2019;2(1):e187223. doi:10.1001/jamanetworkopen.2018.7223

3. Dupont WD, Plummer WD. Power and sample size calculations for studies involving linear regression. *Control Clin Trials*. 1998;19(6):589-601. doi:S0197-2456(98)00037-3 [pii]

4. Walley KR, Thain KR, Russell JA, et al. PCSK9 is a critical regulator of the innate immune response and septic shock outcome. *Sci Transl Med*. 2014;6(258):258ra143-258ra143. doi:10.1126/scitranslmed.3008782

5. Ference BA, Robinson JG, Brook RD, et al. Variation in PCSK9 and HMGCR and Risk of Cardiovascular Disease and Diabetes. *N Engl J Med*. 2016;375(22):2144-2153. doi:10.1056/NEJMoa1604304

© 2019 Feng Q et al. *JAMA Network Open*
